# Different metastatic pattern according to the KRAS mutational status and site-specific discordance of KRAS status in patients with colorectal cancer

**DOI:** 10.1186/1471-2407-12-347

**Published:** 2012-08-09

**Authors:** Mi-Jung Kim, Hye Seung Lee, Jee Hyun Kim, Yu Jung Kim, Ji Hyun Kwon, Jeong-Ok Lee, Soo-Mee Bang, Kyoung Un Park, Duck-Woo Kim, Sung-Bum Kang, Jae-Sung Kim, Jong Seok Lee, Keun-Wook Lee

**Affiliations:** 1 Department of Internal Medicine, Seoul National University Bundang Hospital, Seoul National University College of Medicine, Seongnam, South Korea; 2 Department of Pathology, Seoul National University Bundang Hospital, Seoul National University College of Medicine, Seongnam, South Korea; 3 Department of Internal Medicine, Seoul National University Hospital, Seoul National University College of Medicine, Seoul, South Korea; 4 Department of Laboratory Medicine, Seoul National University Bundang Hospital, Seoul National University College of Medicine, Seongnam, South Korea; 5 Department of Surgery, Seoul National University Bundang Hospital, Seoul National University College of Medicine, Seongnam, South Korea; 6 Department of Radiation Oncology, Seoul National University Bundang Hospital, Seoul National University College of Medicine, Seongnam, South Korea

**Keywords:** KRAS mutation, Lung metastasis, Discordance, Colorectal cancer

## Abstract

**Background:**

We evaluated the association between a KRAS mutational status and various clinicopathologic features including the metastatic pattern in patients with metastatic or recurrent colorectal cancer (MRCRC). The concordance rates of the KRAS status between primary tumor sites and paired metastatic organs were also analyzed.

**Methods:**

The KRAS mutational status in codons 12, 13, and 61 from formalin-fixed sections of both primary tumors and related metastases was determined by sequencing analysis. One hundred forty-three Korean patients with MRCRC with available tissues (resection or biopsy) from both primary tumors and related metastatic sites were consecutively enrolled.

**Results:**

The KRAS mutation rate was 52.4% (75/143) when considering both the primary and metastatic sites. When the relationship between the KRAS status and initial metastatic sites at the time of diagnosis of MRCRC was analyzed, lung metastasis was more frequent as the initial metastatic site in patients with the KRAS mutation than in patients without the KRAS mutation (45.3% vs. 22.1%; *P* = 0.003). However, liver (37.3% vs. 70.6%; *P* < 0.001) or distant lymph node metastases (6.7% vs. 19.1%; *P =* 0.025) were less frequent as the initial metastatic organ in patients with the KRAS mutation than in patients without the KRAS mutation. The discordance rate of KRAS mutational status between primary and paired metastatic sites other than the lung was 12.3% (13/106). Compared with primary tumor sites, the KRAS discordance rate was significantly higher in matched lung metastases [32.4% (12/37)] than in other matched metastatic organs (*P =* 0.005).

**Conclusions:**

Organs initially involved by distant metastasis were different according to the KRAS mutational status in MRCRC patients. The concordance rate (87.7%) of the KRAS mutation status at metastatic sites other than the lung was generally high compared with primary tumor sites; however, lung metastasis had a high rate of KRAS discordance (32.4%).

## Background

Colorectal cancer (CRC) is the most common gastrointestinal cancer, and one of the leading causes of cancer deaths worldwide [1]. Recently, the incidences of CRC have been increasing in Asian countries including Korea [2]. Metastatic or recurrent colorectal cancer (MRCRC) has a poor prognosis. Many recent studies have demonstrated that MRCRC with KRAS mutant-type (MT) are resistant to anti-epidermal growth factor receptor (EGFR) agents such as cetuximab or panitumumab, which showed efficacy as monotherapy or in combination with cytotoxic agents in MRCRC patients with a wild-type (WT) genotype in KRAS codons 12 and 13 [3-6]. KRAS mutational analysis of primary or metastatic tumor tissues is recommended for all MRCRC patients receiving anti-EGFR chemotherapy.

The KRAS mutation is known to occur in about 40% of all CRC cases [7]. The activating mutation of KRAS induces stimulation of the RAS/MAPK signaling pathway independent of EGFR, subsequently followed by dysregulated cell growth, proliferation, and survival [8]. However, the actual differences in clinical behaviors between tumors with WT and MT KRAS, except resistance to anti-EGFR agents, remain controversial. There have been some reports showing that MT KRAS tumors have a poorer prognosis than WT KRAS tumors; however, such a relation between KRAS status and prognosis in CRC was not verified in other reports [9-13]. Recently, it has been reported that the recurrence pattern after the curative resection of CRC is determined according to the KRAS mutational status, showing the positive correlation of KRAS mutation with lung relapse [14,15]. Furthermore, some reports demonstrated a different prognosis and clinical presentation with respect to the mutation types of the KRAS gene [16,17]. However, these findings need to be confirmed by additional studies.

In addition, WT KRAS tumors are not always sensitive to EGFR-targeted therapy. Many studies have focused on the downstream signaling pathways of EGFR with the goal of identifying other predictive factors for patients resistant to anti-EGFR agents. BRAF or PIK3CA mutation and PTEN loss were suggested as other biomarkers predicting a lack of response to anti-EGFR agents [18-21]. Since the BRAF mutation or PTEN loss are rare [22], other mechanisms of resistance to anti-EGFR agents are expected to play a substantial role. Although the KRAS mutation is believed to be an early event in the carcinogenesis of CRC [23] and it has been demonstrated that the KRAS mutational status is highly concordant between primary tumors and related metastases [14,15,24-28], some recent studies have produced contradictory results in regards to the KRAS mutational status [11,29-31]. Especially, most previous studies on the concordance of the KRAS mutation status were conducted on easily available hepatic metastatic tissues [24,26,28]. Therefore, if the discordant rate is different according to the respective metastatic organs, it may also contribute to a poor response to anti-EGFR agents.

Therefore, this study was performed to determine whether there are differences in clinical behavior such as metastatic patterns according to the KRAS mutational status in MRCRC patients. Additionally, the concordance rates of KRAS mutation status between primary sites and respective metastatic organs were also evaluated.

## Methods

### Study population

Among patients with histologically confirmed colorectal adenocarcinoma who had been treated or followed up at medical oncology clinics of Seoul National University Bundang Hospital (SNUBH) between April 2010 and February 2011, 151 MRCRC patients who had undergone surgical resection or biopsy of both primary tumors and related metastatic sites were consecutively enrolled. Eight patients were excluded because of a failure in the process of KRAS mutational analysis on either primary or metastatic site, thus 143 patients were finally included in this study. The clinical data on enrolled patients was retrieved from the CRC database maintained at SNUBH [32], and additional data collection was retrospectively supplemented after a review of the electronic medical record (EMR). This study was approved by the institutional review board of SNUBH.

### Preparation of tumor DNA and sequence analysis of KRAS gene

Formalin-fixed paraffin-embedded primary tumor and metastatic tissue specimens were chosen for each patient and all specimens were microdissected manually under the supervision of experienced gastrointestinal pathologists. After manual microdissection, > 60% of the sample area was shown to contain tumor cells as estimated from the H&E-stained slides. The DNA was extracted using a chelating ion exchange resin (InstaGene Matrix, Bio-Rad). For mutation analyses in codons 12, 13, and 61 of the KRAS gene, extracted tumor DNA samples were subjected to automated sequencing using an ABI 3130xl Genetic Analyzer (Applied Biosystems) and the data was analyzed with GeneMapper Software Version 4.0 (Applied Biosystems). Primer sequences for exon 1 were 5’-AACCTTATGTGTGACATGTTCTA-3’ (forward) and 5’-TGGTCCTGCACCAGTAAT-3’ (reverse); for exon 2, 5’-ACTGTAATAATCCAGACTGTGTT-3’ (forward) and 5’-CCCACCTATAATGGTGAATATCT-3’ (reverse). The polymerase chain reaction (PCR) conditions were as follows: one cycle of 95°C for 5 minutes; 34 cycles of 95°C for 30 seconds, 58°C for 30 seconds, and 72°C for 1 minute; and one cycle of 72°C for 10 minutes.

### Determination of microsatellite instability (MSI) status

The MSI status of paired normal and tumor DNA samples was assessed using the Bethesda panel of 5 microsatellite markers. Sequencing was conducted using the same PCR system as the KRAS mutational analysis. The MSI status was classified as high frequency if ≥ 2 of 5 markers exhibited instability, as low frequency if one marker exhibited instability, and as stable if no markers exhibited instability.

### Statistical analysis

The concordance rate of KRAS mutational status in primary tumors and related metastases was evaluated; the Kappa index was measured using Cohen’s *k*-test, which can assess the concordance between categorical variables of the same individuals. The effect of KRAS mutational status on clinicopathologic parameters and initial metastatic sites were assessed using Pearson’s chi square or Fisher’s exact tests. Multivariate logistic regression analyses were performed to evaluate the effect of other clinicopathologic features on liver-only and lung-only metastases besides the KRAS status. The relationship between the discordant rates of the KRAS mutation status and various clinical parameters including respective metastatic sites was also evaluated using univariate (Pearson’s chi square or Fisher’s exact tests) and multivariate logistic regression analyses. In multivariate logistic regression analysis, the forward stepwise regression model including variables with a *P*-value < 0.10 in univariate analysis was used. Two-sided *P-*values of < 0.05 were considered significant. All analyses were performed using SPSS for Windows, version 17.0 (SPSS Inc., Chicago, IL, USA).

## Results

### Patient characteristics

Of the 143 patients included in this study, 77 patients (53.8%) were male. The median age was 59 years (range, 20–83 years). Paired metastatic sites for KRAS analysis were liver (N = 47), lung (N = 37), peritoneum (N = 30), distant lymph nodes (LN) (N = 17), ovary (N = 11) and pancreas (N = 1), respectively. Most patients had stage IV disease (distant metastasis) at the time of initial CRC diagnosis (N = 101, 70.6%), and only 42 patients had recurred disease after curative resection for localized disease (stage I to III at the time of initial CRC diagnosis). The primary tumor sites were colon in 108 patients (76.5%) and rectum in 35 (23.5%). Other patient characteristics are shown in Table 1.

**Table 1:** Patient characteristics (N = 143) and the association between KRAS mutational status and clinicopathologic parameters

**Characteristic**	**No. of patients**			
	**All**	**KRAS WT**	**KRAS MT**	***P*-value**
	**N**	**N (%)**	**N (%)**	
Gender				0.897
Male	77	37 (54.4)	40 (53.3)	
Female	66	31 (45.6)	35 (46.7)	
Age				0.303
< 65 years	97	49 (72.1)	48 (64.0)	
≥ 65 years	46	19 (27.9)	27 (36.0)	
Clinical situations for the development of systemic metastasis				0.010
Stage IV at the time of initial diagnosis	101	55 (80.9)	46 (61.3)	
Recurred after curative resection	42	13 (19.1)	29 (38.7)	
Primary site				0.802
Colon	108	52 (76.5)	56 (74.7)	
Rectum	35	16 (23.5)	19 (25.3)	
Histology and grade				0.819
ADC, well differentiated	5	2 (2.9)	3 (4.0)	
ADC, moderately differentiated	118	56 (82.4)	62 (82.7)	
ADC, poorly differentiated	12	7 (10.3)	5 (6.7)	
ADC, mucinous	8	3 (4.4)	5 (6.7)	
Gross morphology of primary tumor				0.421
Ulceroinfiltrative	66	33 (48.5)	33 (44.0)	
Ulcerofungating	63	28 (41.2)	35 (46.7)	
Polypoid	7	2 (2.9)	5 (6.7)	
Unknown	7	5 (7.4)	2 (2.7)	
Obstruction of primary tumor				0.371
Yes	43	18 (26.5)	25 (33.3)	
No	100	50 (73.5)	50 (66.7)	
Perforation of primary tumor				1.000
Yes	10	5 (7.4)	5 (6.7)	
No	133	63 (92.6)	70 (93.3)	
Microsatellite instability				0.343
Stable	121	58 (85.3)	63 (84.0)	
Low frequency	11	3 (4.4)	8 (10.7)	
High frequency	2	1 (1.5)	1 (1.3)	
Unknown	9	6 (8.8)	3 (4.0)	

Abbreviations: WT, wild-type; MT, mutant-type; ADC, adenocarcinoma.

### Frequency and types of KRAS mutation

The KRAS mutation was observed in 62 (43.4%) primary tumors and at 63 (44.1%) related metastatic sites. Seventy-five patients (52.4%) had the KRAS mutation in any place of the primary or metastatic sites. Of those 75 patients, 20 patients had a Gly12Asp mutation, 18 had a Gly13Asp mutation, 17 had a Gly12Val mutation, 9 had a Gly12Cys, 3 had a Gly12Ala, and other 3 patients had a Gly12Ser. In addition, 2 patients had a Gln61Leu mutation and Gly13Cys (N =1), Gln61His (N = 1), and Gln61Arg mutation (N = 1) were detected in the remaining 3 patients (Table 2). The incidence of codon 61 mutations was 5.3% among patients with the KRAS mutation (4 of 75 cases).

**Table 2:** Distribution of KRAS mutation types

**Mutation types**	**N**	**%**
Gly12Asp	20	26.7
Gly12Val	17	22.7
Gly12Cys	9	12.0
Gly12Ala	3	4.0
Gly12Ser	3	4.0
Gly13Asp	18	24.0
Gly13Cys	1	1.3
Gln61Leu	2	2.7
Gln61His	1	1.3
Gln61Arg	1	1.3

### Concordance of KRAS status in primary tumors and related metastases

KRAS status was concordant between primary and metastatic sites in 118 patients (82.5%; kappa = 0.645) (Table 3). Of the 25 discordant cases, 12 patients had the KRAS mutation in the primary tumors, and not in the metastatic sites; 13 patients had the KRAS mutation in the metastatic tumors, and not in the primary tumors. We analyzed the difference in discordance pattern according to clinical presentation (stage IV vs. recurred) at the time of initial MRCRC diagnosis and metastatic organs from which tissue specimens were obtained. There were no statistically significant differences in discordance patterns with respect to these parameters (Table 4).

**Table 3:** KRAS mutational status of primary tumors and paired metastatic sites

**KRAS status**	**No. of WT KRAS (P)**	**No. of MT KRAS (P)**
No. of WT KRAS (M)	68	12^a^
No. of MT KRAS (M)	13^a^	50

^a^ Discordant cases.

Abbreviations: WT, wild-type; P, primary tumors; MT, mutant-type; M, paired metastatic sites.

**Table 4:** Analyses of KRAS discordance patterns in KRAS discordant cases (N = 25)

**Characteristic**	**WT (P) → MT (M)**	**MT (P) → WT (M)**	**Total N**	***P*-value**
	**N (%)**	**N (%)**		
Clinical situations for the development of systemic metastasis				0.411
Stage IV at the time of initial diagnosis	7 (43.8)	9 (56.3)	16	
Recurred after curative resection	6 (66.7)	3 (33.3)	9	
Analyzed metastatic sites				0.377
Liver	1 (20.0)	4 (80.0)	5	
Lung^a^	6 (50.0)	6 (50.0)	12	
Distant lymph nodes	1 (50.0)	1 (50.0)	2	
Peritoneum	3 (75.0)	1 (25.0)	4	
Ovary	2 (100.0)	0 (0.0)	2	

Abbreviations: WT, wild-type; P, primary tumors; MT, mutant-type; M, paired metastatic sites.

^a^ There was no statistically significant difference in the KRAS discordance pattern between the lung and other paired metastatic organs (*P* = 1.000).

### Association between KRAS mutational status and clinicopathologic features

Clinical situations for the development of systemic metastasis in MRCRC patients were different according to the KRAS mutational status. In regards to the development of distant metastasis, systemic relapse from previously localized CRC (stages I to III) after complete surgical resection was more frequent in MT KRAS patients than in WT KRAS patients (38.7% vs. 19.1%); however, systemic metastasis was more frequent at the time of initial CRC diagnosis in WT KRAS patients than in MT KRAS patients (80.9% vs. 61.3%; *P* = 0.010).

There was no association between the KRAS status and other clinicopathologic features (gender, age, primary tumor location, histology, gross morphology, obstruction or perforation of primary tumors, and MSI) (Table 1).

### KRAS mutational status and metastatic patterns

When the relationship between KRAS status and initial metastatic sites at the time of diagnosis of stage IV disease or recurrence (in the cases of initial stages I to III) was analyzed (N = 143), patients with MT KRAS had a higher rate of initial lung metastasis (45.3%) than patients with WT KRAS (22.1%; *P =* 0.003). However, a higher percentage of liver or distant LN metastases was detected in patients with WT KRAS when compared to patients with MT KRAS (70.6% vs. 37.3%, *P* < 0.001 for liver; 19.1% vs. 6.7%, *P* = 0.025 for LN). Peritoneal or ovarian metastases showed no significant difference according to the KRAS mutational status (*P*-values > 0.05) (Table 5). Regarding other initial metastatic sites including bone (N = 3), kidney (N = 2) and so on, comparison could not be made due to too few cases.

**Table 5:** **Association between initial metastatic or recurred sites and KRAS mutational status (N = 143)**^**a**^

**Initial metastatic or recurred site**	**WT KRAS**	**MT KRAS**	**Total N**	***P*-value**
	**N (%)**	**N (%)**		
Liver				< 0.001
Yes	48 (70.6)	28 (37.3)	76	
No	20 (29.4)	47 (62.7)	67	
Lung				0.003
Yes	15 (22.1)	34 (45.3)	49	
No	53 (77.9)	41 (54.7)	94	
Distant lymph nodes				0.025
Yes	13 (19.1)	5 (6.7)	18	
No	55 (80.9)	70 (93.3)	125	
Peritoneum				0.451
Yes	17 (25.0)	23 (30.7)	40	
No	51 (75.0)	52 (69.3)	103	
Ovary^b^				0.885
Yes	5 (7.4)	6 (8.0)	11	
No	63 (92.6)	69 (92.0)	132	

^a^ Initial metastatic or recurred sites were defined as the organs involved by distant metastasis at the time point of diagnosis of stage IV cancer (initial stage IV disease) or recurrence with distant metastasis (recurred cases from initial stage I-III disease). In these analyses, all enrolled cases (N = 143) were included.

^b^All cases who had Krukenberg tumors as the initial metastatic or recurred sites had peritoneal metastasis simultaneously. There were no cases with ovarian metastasis alone without peritoneal metastasis in our study.

Abbreviations: WT, wild-type; MT, mutant-type.

KRAS mutational status in patients with initial distant metastasis confined to a single organ at the time of diagnosis of stage IV or recurred disease was also analyzed (N = 113). A higher rate of lung-only metastasis was observed in MT KRAS cases (41.0%) compared with WT KRAS cases (11.5%; *P* < 0.001). Liver-only metastasis was more frequently observed in WT KRAS cases (65.4%) than in MT KRAS cases (27.9%; *P* < 0.001). However, the frequencies of developing metastasis only confined to distant LN, peritoneum or other organs were not significantly different between patients with WT and MT KRAS (Table 6).

**Table 6:** **Analysis of metastatic patterns among patients with single organ-only metastasis (N = 113)**^**a**^

**Initial metastatic or recurred site**	**WT KRAS**	**MT KRAS**	**Total N**^**a**^	***P*-value**
	**N (%)**	**N (%)**		
Liver				< 0.001
Yes	34 (65.4)	17 (27.9)	51	
No	18 (34.6)	44 (72.1)	62	
Lung				< 0.001
Yes	6 (11.5)	25 (41.0)	31	
No	46 (88.5)	36 (59.0)	82	
Distant lymph nodes				0.411
Yes	4 (7.7)	2 (3.3)	6	
No	48 (92.3)	59 (96.7)	107	
Peritoneum^b^				0.062
Yes	7 (13.5)	17 (27.9)	24	
No	45 (86.5)	44 (72.1)	89	

^a^ The association between initial metastatic or recurred sites and KRAS status was analyzed in patients with metastasis confined to a single organ at the time of diagnosis of MRCRC (N = 113; 30 patients with initial systemic metastasis involving ≥ 2 organs simultaneously at the time of diagnosis of MRCRC were excluded).

^b^ Of 24 patients, cases with simultaneous peritoneal and ovarian metastases (N = 6) were included; there were no cases with ovarian metastasis alone without peritoneal metastasis in our study. When patients with peritoneal metastasis only without Krukenberg tumor (N = 107) were separately analyzed, the result was not different [WT KRAS 10.0% (5/50) vs. MT KRAS 22.8% (13/57); *P* = 0.077].

Abbreviations: WT, wild-type; MT, mutant-type; MRCRC, metastatic or recurrent colorectal cancer.

Analyses of the clinical predictive factors for liver- or lung-only metastasis in patients with initial distant metastasis confined to a single organ (N = 113) were performed including other clinical variables [gender, age (< 65 vs. ≥ 65 years), clinical situations for the development of systemic metastasis (stage IV at the time of initial CRC diagnosis vs. recurred after curative resection of stage I - III CRC), primary tumor sites (colon vs. rectum), histologic grade, MSI, etc.] along with the KRAS status. Multivariate logistic regression analysis was carried out using variables with *P-*values *<* 0.10 in univariate analysis. KRAS status remained predictive for both liver-only and lung-only metastases [odds ratio (OR) = 0.24; 95% confidence interval (CI), 0.10 – 0.55; *P* = 0.001 for liver-only metastasis and OR = 4.20; 95% CI, 1.43 – 12.33; *P* = 0.009 for lung-only metastasis, respectively] in the multivariate analysis. Recurred cases had increased risk of developing lung-only metastasis and decreased risk of developing liver-only metastasis compared with initial stage IV cases. Rectal cancer patients had increased risk of developing lung-only metastasis compared with colon cancer patients; however, the primary tumor location was not predictive for liver-only metastasis in the multivariate analysis (Table 7).

**Table 7:** **Multivariate logistic regression analysis on the correlation between clinical parameters including KRAS status and liver- or lung-only metastases**^**a**^

**Clinical parameter**	**Liver-only metastasis**		**Lung-only metastasis**	
	**OR (95% CI)**	***P***	**OR (95% CI)**	***P***
KRAS status (MT vs. WT)	0.24 (0.10-0.55)	0.001	4.20 (1.43-12.33)	0.009
Tumor site (rectum vs. colon)	-	-	3.32 (1.15-9.59)	0.026
Clinical situations for the development of systemic metastasis (recurred vs. stage IV)	0.22 (0.085-0.57)	0.002	6.24 (2.32-16.77)	< 0.001

^a^ In this analysis, only variables which had *P-*values *<* 0.10 in univariate analysis were included in patients with single organ-only metastasis (N = 113).

Abbreviations: OR, odds ratio; CI, confidence interval; MT, mutant-type; WT, wild-type.

### Discordance rates of KRAS status according to the respective metastatic sites

We evaluated the discordance rates of KRAS mutation status between primary tumors and paired tissues from various metastatic organs (Table 8). The lung was the most frequent site showing KRAS discordance (32.4%; 12/37); however, the discordant rate at metastatic sites other than the lung was 12.3% (13/106) (*P* = 0.005; Table 8). When the discordance rates of the KRAS status between primary tumors and respective metastatic sites (lung, peritoneum, distant LN or ovary) were compared with the discordance rate between primary tumors and hepatic metastatic tissues (10.6%), pulmonary metastasis only showed a significantly higher discordance rate of KRAS mutation status (32.4%; *P =* 0.014). However, no difference in the discordance rate of KRAS status for the other metastatic organs [peritoneum (13.3%), LN (11.8%), or ovary (18.2%)] was observed relative to liver metastasis (*P*-values > 0.05; Table 8). One patient with tissues acquired from a primary tumor (colon) and related pancreatic metastatic site showed concordance of KRAS status (KRAS mutant; Gly12Cys).

**Table 8:** **The discordant rates of KRAS mutation status between primary tumors and respective metastatic sites**^**a,b**^

	**Concordant cases, N (%)**	**Discordant cases, N (%)**	**Total N**	***P*-value**
Liver	42 (89.4)	5 (10.6)	47	-
Lung	25 (67.6)	12 (32.4)	37	0.014
Peritoneum	26 (86.7)	4 (13.3)	30	0.730
Distant lymph nodes	15 (88.2)	2 (11.8)	17	1.000
Ovary	9 (81.8)	2 (18.2)	11	0.607

^a^ The discordant rates of KRAS mutation status between primary tumors and respective metastatic sites (lung, peritoneum, distant lymph nodes and ovary) were compared with the discordant rate of KRAS status between primary tumor and hepatic metastatic sites (10.6%) using the Pearson’s chi square or Fisher’s exact tests.

^b^ The KRAS discordant rate of lung metastasis (32.4%; 12/37) was also significantly higher when compared with the discordant rate of combining all other metastatic organs [12.3%; 13/106 (*P* = 0.005)].

Additional analyses were performed to reaffirm whether the discordance rates of KRAS status were influenced by other various clinicopathologic factors such as primary tumor location, type of primary or metastatic tumor specimens (biopsied vs. resected), and the presence of chemotherapy (± radiotherapy) before obtaining primary or metastatic tumor specimens, along with the metastatic organs. In univariate analyses, rectal cancers showed an increased KRAS discordance rate when compared with colon cancers (28.6% vs. 13.9%; *P* = 0.047), and biopsied primary tumor specimens showed a trend for an increased discordant rate when compared with resected primary tumor specimens (40.0% vs. 15.8%; *P* = 0.073). However, the site of metastatic organs (lung vs. liver) only affected discordant KRAS status in multivariate logistic regression analysis (OR = 4.03; 95% CI, 1.27 – 12.80; *P* = 0.018) (Table 9).

**Table 9:** **Univariate and multivariate analyses on the association between clinical parameters and the discordance rates of KRAS mutation status**^**a**^

**Characteristic**	**Univariate analysis**			**Multivariate analysis**	
	**Concordant cases, N (%)**	**Discordant cases, N (%)**	***P***	**OR (95 % CI)**	***P***
Metastatic site			0.019		
Liver	42 (89.4)	5 (10.6)		1 (Referent)	
Lung	25 (67.6)	12 (32.4)		4.03 (1.27-12.80)	0.018
Others	51 (86.4)	8 (13.6)		1.32 (0.40-4.33)	0.649
Tumor site			0.047		
Colon	93 (86.1)	15 (13.9)		-	
Rectum	25 (71.4)	10 (28.6)		-	-
Type of primary tumor specimens			0.073		
Resected	112 (84.2)	21 (15.8)		-	
Biopsied	6 (60.0)	4 (40.0)		-	-

^a^ In multivariate analysis, only variables which had *P-*values *<* 0.10 in univariate analysis were included.

Abbreviations: OR, odds ratio; CI, confidence interval.

## Discussion

To the best of our knowledge, this is the first large-scale study in which a KRAS mutational analysis was performed between primary tumors and corresponding metastases in Asian MRCRC patients. Overall, a high concordance rate of KRAS status was observed in Korean MRCRC patients, as had been previously reported in Western populations. We observed significant differences in initial metastatic patterns according to the KRAS mutational status. MT KRAS tumors developed lung metastases more frequently as the initial metastatic site; however, liver and distant LN were more frequently involved as the initial metastatic sites in WT KRAS tumors. Additionally, the degree of concordance in KRAS mutational status was significantly different according to the sites of related metastatic organs, where the lung was the most frequent metastatic site showing the discordance of KRAS status.

Our study demonstrated that the clinical presentation of CRC varied according to the KRAS mutational status. KRAS mutational status was shown to affect the presenting pattern of distant metastasis in MRCRC patients. Recurrent cases after curative treatment for localized diseases were more common in MT KRAS patients; however, systemic metastasis was more frequent at the time of initial CRC diagnosis in WT KRAS patients (Table 1). One previous study showed that the risk of recurrence was significantly higher for MT KRAS than WT KRAS tumors in patients with localized CRC [10]. If localized CRC patients with MT KRAS had more chances of recurrence, then the MT KRAS cases would be selected and thus a higher frequency of MT KRAS in recurrent CRC patients would be expected than in patients with stage IV disease at the time of initial CRC diagnosis. However, as our patient cohort is relatively small, our assumption needs to be further investigated in future large studies.

In the present study, organs initially involved by distant metastasis were shown to be different according to the KRAS mutational status. Patients with MT KRAS had an initial lung metastasis more frequently than patients with WT KRAS. In contrast, the WT KRAS patients had liver or distant LN metastases more frequently as the initial metastatic sites. However, other metastatic sites such as the peritoneum were not affected by KRAS status (Table 5). When the analyses were conducted on patients with initial distant metastasis confined to a single organ, the results were similar except for distant LN metastasis (Table 6). However, since the number of cases with LN-only metastasis was small (N = 6), the difference of percentages (7.7% vs. 3.3%) might not have reached statistical significance. Although the MT KRAS tumors showed a trend for more frequent development of peritoneum-only metastasis (13.5% vs. 27.9%; *P* = 0.062), it was not statistically significant (Table 6). In the multivariate analysis, KRAS status, primary tumor site, and clinical situations for the development of systemic metastasis were significant predictors for liver-only and/or lung-only metastases (Table 7). The reason why the clinical situations of developing systemic metastasis influenced the initially involved metastatic organs (liver or lung) is not clear; this may be related to the process of patient enrollment to this study as patients with available tissues from both primary and paired metastatic sites were only included. During this process, recurred CRC patients with tissue-available lung metastasis might be selectively included. However, even after adjusting for these clinical variables, the KRAS mutational status was an independent predictive factor for both liver-only and lung-only metastases in our study. A previous study, which analyzed the KRAS status in primary tumors of CRC patients, showed that there were more MT KRAS tumors in patients with lung metastasis than in patients with liver metastasis [14]. Based on this finding, they suggest that KRAS-mutated primary CRC tumors can recur with lung metastasis more frequently than with liver metastasis. Although the KRAS mutational status was regarded as positive if KRAS was mutated in any place of primary tumors or related metastases in the present report, our results also support their suggestion. Furthermore, when the analysis was conducted based on the KRAS status of primary tumor, the result of our study was also the same as above. Our work along with previous studies strongly suggests that the sequence of organs involved by systemic metastasis is influenced by KRAS mutational status in CRC patients.

Our results are generally consistent with previous studies that have reported a high concordance rate of KRAS mutation (about 90%) between primary and metastatic tumors [14,15,24-28]. Paired metastatic tissues in previous studies were mostly derived from the liver [24,26,28] because these tissues were easily available from hepatic metastasectomy [33,34]. However, reports on the degree of KRAS concordance of other metastatic organs other than the liver have been very limited. Our study evaluated the KRAS status of metastatic tissues from various organs besides liver. The degree of KRAS mutational concordance was different according to the related metastatic sites, with a significantly higher rate of discrepancy in lung metastases (32.4%) when compared with other metastatic organs (12.3%) or liver (10.6%) (*P*-values < 0.05; Table 8). Although some studies have recently demonstrated a relatively high degree of discordance, with a discordance rate of up to 50% [11,29-31], there has been no report showing the site-specific KRAS discordance, as was shown in this study. The mechanism behind the discordant KRAS mutational status is still not exactly known [31,35,36]. Sampling errors, heterogeneity within primary tumors, and the development of mutations during the process of metastasis may be the causes of this discordance. The reason why lung is the most frequent site where the KRAS discordance takes place is also unknown. In the present study, we analyzed the pattern of KRAS discordance (i.e. from WT in primary tumors to MT in related metastatic sites or vice versa); as the sample size was small (N = 25), the KRAS discordance pattern did not show any relation to clinical situations for the development of systemic metastasis (stage IV vs. recurred) or metastatic organs from which tumor specimens were obtained. Among 12 cases with KRAS-discordant lung metastasis, 6 cases (50%) had the change of KRAS status from WT in a primary tumor to MT in the lung and 6 cases (50%) had vice versa; there was no statistically significant difference in the discordance pattern between the lung and other paired metastatic organs (Table 4). Therefore, the underlying causes of KRAS discordance need to be further evaluated in future large studies.

Our study has some limitations. First, this study was performed at a single institution and all MRCRC patients diagnosed at our institution were not included. Instead, only MRCRC patients with both primary and paired metastatic tissues were consecutively included. In such a process, unrecognized biases might have influenced our study. Second, a KRAS mutational analysis was not repetitively conducted in cases with discordant KRAS status partly because of insufficient remaining tissue specimens for further examination. This might raise concerns about the sensitivity of the KRAS mutation analysis. We actually used traditional sequencing (Sanger) method with relatively low sensitivity for KRAS mutation analysis [37]. In addition, biopsied specimens of primary tumor showed a trend for higher discordance rate than resected specimens in this study (40.0% vs. 15.8%; *P* = 0.073), although no significance was shown in multivariate analysis. However, we used tumor cell enrichment by microdissection under the supervision of experienced pathologists to increase the sensitivity of the sequencing method. In addition, biopsy of primary tumors in our study was all performed by endoscopy. Actually, a biopsy of distant metastasis may be more problematic than endoscopic biopsy of primary tumors in context of tumor cell percentage [38]. In the present study, a small number of metastatic specimens (8/143) was obtained from needle biopsy and only 1 case had KRAS discordance [1/8 (12.5%) for biopsied metastatic specimens vs. 24/135 (17.8%) for resected metastatic specimens; *P* = 1.000]. Moreover, the concordance rates observed between primary and metastatic tissues including the liver only (89.4%) or all metastatic organs other than lung (87.7%) were similar to the concordance rates reported in previous studies. All these findings suggest that the high KRAS discordant rate of lung metastasis (32.4%) had not simply resulted from types of tumor tissue specimens (biopsied vs. resected) or less sensitive analytic methods performed at our institute. Instead, the results from our study reflect the real situation of clinical fields as the traditional sequencing (Sanger) analysis is the most frequent method used in the real clinical practice setting. More sensitive methods, such as real-time PCR for KRAS mutation analysis, are only used in the investigational setting and not widely spread in the clinical practice.

Despite these limitations, our study provides some clinically meaningful suggestions. The present study demonstrated that the KRAS mutational status was an independently predictive factor for organs initially involved by distant metastasis. This observation implies that surveillance strategies after curative surgery might be tailored to individual CRC patients according to the KRAS mutational status. Postoperative surveillance might be more focused on lung metastasis (i.e., chest computed tomography) in patients with MT KRAS than in patients with WT KRAS, when considering the chance of performing metastasectomy after the early detection of pulmonary metastasis. Our study also raised the hypothesis that the discordant rates of KRAS mutational status might be metastatic site-specific in CRC. Using the sequencing method, we found different discordant rates according to the metastatic sites. A high KRAS discordant rate in patients with lung metastasis, observed in our study, warrants further large validation studies.

## Conclusions

The concordance rate of KRAS mutation in metastatic sites was generally high compared with primary tumor sites in Korean MRCRC patients, as had been previously reported in Western patients. Organs initially involved by distant metastasis were different according to the KRAS mutational status. Individually tailored postoperative surveillance strategies after curative CRC surgery according to the KRAS mutational status need to be further investigated in future studies. In addition, lung metastasis had a higher rate of KRAS discordance (32.4%) than other metastatic organs, suggesting a possibility of site-specific KRAS discordance in MRCRC patients. This observation should be clarified in further large studies.

## End notes

This article was partly presented at the European Multidisciplinary Cancer Congress, Stockholm, Sweden, 23-27 September 2011.

## Competing interests

The authors declare that they have no competing interests.

## Authors’ contributions

MJK carried out patient data collection and statistical analysis and drafted this manuscript. HSL and KUP participated in sequencing analysis, data collection and contributed to the interpretation of the data. JHK, YJK, DWK, SBK and JSK treated many of the enrolled patients and contributed to the interpretation of data. JHK and JOL assisted patient data collection and the statistical analysis of the study. SMB and JSL contributed substantially to the interpretation of data, and helped the statistical analysis. KWL conceived this study, participated in its design and coordination, treated many of enrolled patients, and helped to draft the manuscript. All authors read and approved the final manuscript.

## Pre-publication history

The pre-publication history for this paper can be accessed here:

http://www.biomedcentral.com/1471-2407/12/347/prepub
